# Development and Pilot in Vivo Testing of a Protocol for PLGA–Vancomycin Coatings on PTFE Used as Silicone-Implant Analogs

**DOI:** 10.3390/medicina62010081

**Published:** 2025-12-30

**Authors:** Alina-Alexandra Negrilă, Oliviu Nica, Maria Viorica Ciocîlteu, Andrei Bită, Claudiu Nicolicescu, Alexandru-Bogdan Popescu, Marius-Eugen Ciurea

**Affiliations:** 1 Department of Plastic Surgery, Faculty of Medicine, University of Medicine and Pharmacy of Craiova, 200349 Craiova, Romania; 2 Department of Instrumental and Analytical Chemistry, Faculty of Pharmacy, University of Medicine and Pharmacy of Craiova, 200349 Craiova, Romania; maria.ciocilteu@umfcv.ro; 3 Department of Pharmacognosy & Phytotherapy, Faculty of Pharmacy, University of Medicine and Pharmacy of Craiova, 200349 Craiova, Romania; 4 Department of Engineering and Management of Technological Systems, Faculty of Mechanics, University of Craiova, 220037 Drobeta Turnu-Severin, Romania; 5 Faculty of Pharmacy, University of Medicine and Pharmacy of Craiova, 200349 Craiova, Romania

**Keywords:** Polylactic-Co-Glycolic acid, rats, coated materials, drug delivery systems, subcutaneous tissue

## Abstract

*Background and Objectives*: Implant-associated complications, including foreign-body responses and infection risk, remain major concerns in reconstructive and aesthetic breast surgery. Antimicrobial polymer coatings have been proposed as potential preventive strategies, but early-stage development requires simple and ethically refined in vivo models. This pilot study aimed to (i) establish a practical workflow for applying PLGA–vancomycin coatings onto PTFE substrates used as experimental analogs for smooth silicone implants, and (ii) develop a small-animal implantation protocol for short-term evaluation of surgical feasibility and local tissue tolerability. *Materials and Methods*: PLGA microparticles and PLGA–vancomycin microparticles were prepared using a double-emulsion solvent-evaporation method and applied onto PTFE discs. Particle size and polydispersity were assessed based on dynamic light scattering (DLS), and surface charge was measured via zeta potential. A bilateral subcutaneous implantation model was used in four Wistar rats, each receiving a PTFE disc coated with PLGA-only on one side and a disc coated with PLGA–vancomycin on the other. Animals were monitored for postoperative recovery, wound appearance, and general condition. After four weeks, implants and surrounding tissues were harvested for macroscopic and preliminary histological evaluation. *Results*: Both PLGA-only and PLGA–vancomycin microparticles showed submicron mean hydrodynamic diameters and moderately polydisperse distributions typical for double-emulsion formulations. All animals recovered normally, maintained stable body weight, and exhibited no macroscopic signs of adverse reactions. Preliminary histology showed early fibrous capsule formation with mild inflammatory infiltrate around both types of coated implants, without qualitative differences observed in this pilot setting. *Conclusions*: This preliminary study demonstrates the feasibility of applying PLGA-only and PLGA–vancomycin coatings onto PTFE implant analogs and establishes a reproducible, minimal-use rat model for short-term evaluation of local tissue tolerability. The protocol provides a practical foundation for future work on coating stability, drug-release kinetics, antibacterial activity, and long-term tissue responses on medical-grade silicone substrates.

## 1. Introduction

Postoperative complications remain a significant challenge in reconstructive and aesthetic breast surgery, particularly those related to implant-associated inflammation, infection, and long-term foreign-body responses. Surgical site infections represent a serious early complication, while capsular contracture—an inflammatory and fibrotic reaction surrounding the implant surface—can compromise aesthetic results, cause pain and deformity, and often necessitates revision surgery, with substantial impact on patient quality of life and healthcare costs [1,2].

Capsular contracture is driven by inflammatory signaling, fibroblast activity, extracellular matrix remodeling, and biofilm-related chronic stimuli [3,4,5,6,7,8]. These mechanisms provide essential biological context for implant-associated complications, although they were not evaluated in this pilot study. *Staphylococcus aureus* and *Staphylococcus epidermidis*, major biofilm-forming pathogens in breast implant infections [7], can maintain low-grade inflammation that is poorly addressed by systemic antibiotics due to limited penetration at the implant–tissue interface [9]. This has encouraged the development of localized, con-trolled-release antimicrobial systems [8].

Poly(lactic-co-glycolic acid) (PLGA) has emerged as a versatile polymer for localized drug delivery. It is biodegradable, approved by the U.S. Food and Drug Administration (FDA), and is capable of encapsulating both hydrophilic and hydrophobic compounds [10,11,12,13]. PLGA-based systems can provide prolonged release kinetics and have been explored as multifunctional coatings capable of modulating local tissue responses and reducing bacterial colonization [14]. Vancomycin, widely used for treating Gram-positive infections including MRSA, exhibits limited tissue penetration and a short systemic half-life, making it an excellent candidate for localized delivery [15,16,17]. Double-emulsion solvent-evaporation (W1/O/W2) is a well-established technique for encapsulating hydrophilic drugs such as vancomycin within PLGA microparticles [18], and PLGA coatings have been shown to support drug adhesion and sustained release on various implantable substrates [19]. Additional hybrid organic–inorganic coating systems have been developed to improve mechanical and chemical stability in biomaterials [20].

Despite growing interest in PLGA-based antimicrobial coatings, there is currently no simple and reproducible small-animal model suitable for early in vivo evaluation of such coatings on smooth implant-like materials relevant to breast surgery. Existing models frequently rely on in vitro assays or materials that do not approximate silicone surfaces, leaving a methodological gap for early, ethically refined feasibility testing.

In this context, PTFE was selected as an experimental analog for silicone implants due to its smooth surface, chemical inertness, mechanical stability, and ability to form a reproducible foreign-body capsule in rodents [21]. PTFE maintains its structural integrity during implantation and is practical for small-animal surgery. While PTFE does not replicate all physicochemical characteristics of clinical silicone, it provides a suitable substrate for early workflow development and feasibility assessment.

The present work was intentionally designed as a protocol-oriented pilot study with a deliberately limited scope. Its primary objectives were:

(1) to develop a practical and reproducible workflow for applying PLGA-only and PLGA–vancomycin coatings onto PTFE implant analogs, and
(2) to establish a simplified bilateral rat implantation model for short-term evaluation of surgical feasibility and local tissue tolerability.

This pilot study focuses exclusively on procedural feasibility and early local tissue response. It does not assess antibacterial activity, drug-release kinetics, cytokine expression, or long-term capsule maturation; these aspects will be addressed in subsequent stages of the project. By using an intraindividual (paired bilateral) design, each animal received both coating variants, reducing intersubject variability and minimizing animal use in accordance with ethical guidelines.

## 2. Materials and Methods

### 2.1. Materials

Poly(lactic-co-glycolic acid) (PLGA, 65:35 lactide:glycolide ratio; Sigma-Aldrich, St. Louis, MO, USA) was used as the biodegradable polymer matrix [13]. Vancomycin hydrochloride (Sigma-Aldrich) served as the model hydrophilic antibiotic [15]. Dichloromethane (DCM; analytical grade, Merck, Darmstadt, Germany) was used as the organic solvent. Poly(vinyl alcohol) (PVA, 88% hydrolyzed; Sigma-Aldrich) acted as a stabilizing surfactant. Ultrapure water (Milli-Q system, Millipore, Bedford, MA, USA) was used throughout the experiments. All chemicals were used as received, without further purification.

### 2.2. Preparation of Vancomycin-Loaded PLGA Microparticles

Vancomycin-loaded PLGA microparticles were synthesized using the double-emulsion solvent-evaporation (W1/O/W2) technique [18]. In this pilot study, the internal aqueous phase contained 50 mg of vancomycin; however, no analytical determination (HPLC or UV-Vis) was performed to quantify the amount of drug encapsulated in the PLGA microparticles, as the focus was on establishing the coating workflow and in vivo model. An internal aqueous phase (W1, 5 mL), consisting of 0.5% (*w*/*v*) PVA and 50 mg of vancomycin, was prepared via complete dissolution of the antibiotic. The organic phase (O) contained 100 mg of PLGA dissolved in 2 mL of dichloromethane (DCM) under gentle stirring until a homogeneous solution was obtained. The W1 phase was added dropwise into the organic phase under vigorous mixing (50,000 rpm for 2 min) using a SilentCrusher vortex homogenizer to form a water-in-oil (W1/O) primary emulsion.

**Figure 1:**
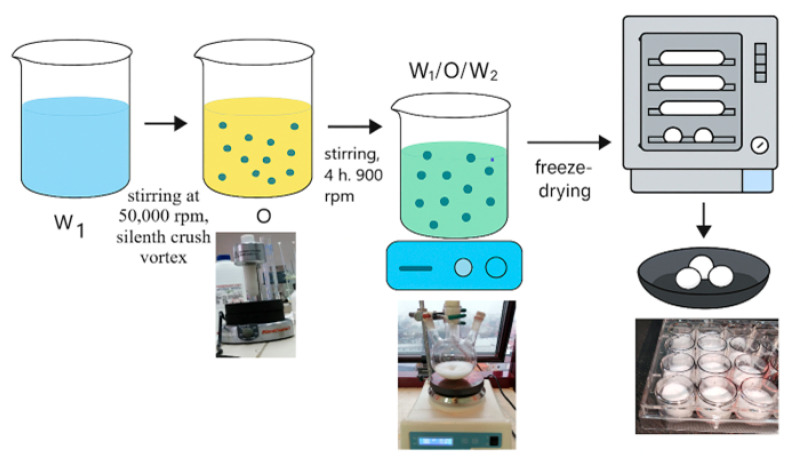
PLGA–antibiotic delivery system formulation, W/O/W double emulsion followed by lyophilization. O: Oil; W: Water.

The primary emulsion was then transferred into an external aqueous phase (W2, 100 mL of 0.5% PVA) under continuous stirring at 900 rpm. The resulting double emulsion (W1/O/W2) was maintained under agitation for approximately 4 h at room temperature to promote solvent evaporation and solidification of the PLGA matrix, entrapping the antibiotic within [8,18]. The resulting suspension was centrifuged at 11,000 rpm for 10 min to collect the microparticles. The pellet was washed three times with ultrapure water to remove unbound drug and residual PVA. For drying and storage, the washed particles were resuspended in a small volume of water and subjected to lyophilization, yielding a fine, dry, porous PLGA–vancomycin powder. The final product was stored at −8 °C until use (Figure 1).

### 2.3. Preparation of Blank PLGA Microparticles (Negative Control)

Blank (drug-free) PLGA microparticles were prepared using the same protocol, except that the internal aqueous phase contained only 5 mL of 0.5% (*w*/*v*) PVA solution without vancomycin.

Although such particles are typically used as controls in in vitro release or antibacterial assays, these evaluations were not part of the present pilot study and were not performed here.

### 2.4. Particle Size, Zeta Potential, and Polydispersity

The hydrodynamic diameter, zeta potential, and polydispersity index (PDI) of PLGA-only and PLGA–vancomycin microparticles were measured using dynamic light scattering (DLS) using a Brookhaven 90PLUS/BI-MAS zeta potential analyzer (Brookhaven Instruments, Nashua, NH, USA). Measurements were performed at 25 °C using ultrapure water as the dispersant (refractive index 1.330, viscosity 0.8872 cP).

Microparticles were resuspended in filtered deionized water at a 1:20 (*w*/*v*) dilution and gently sonicated in a water-bath sonicator for 2 min to promote uniform dispersion prior to measurement. For each formulation, measurements were performed in triplicate, with each reading obtained from an independently prepared particle suspension. Data were reported as mean ± standard deviation.

### 2.5. Coating of PTFE Discs

PTFE discs (10 mm diameter, 2 mm thickness) were used as experimental analogs for smooth silicone implants due to their inert, hydrophobic surface and their established use in subcutaneous rodent models [21]. PTFE does not replicate all physicochemical characteristics of medical-grade silicone; however, it provides a practical substrate for early feasibility testing due to its low frictional coefficient and dielectric constant, thermal stability and chemical inertness, hydrophobicity, and resistance [22].

**Figure 2:**
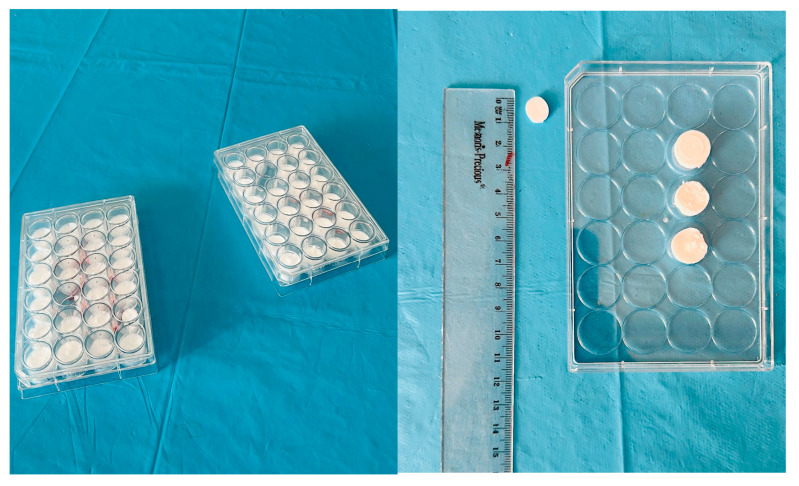
The PLGA–vancomycin-coated PTFE implants.

Before coating, discs were ultrasonically cleaned in ethanol for 15 min, rinsed with ultrapure water, and sterilized at 120 °C for 30 min. For coating preparation, a suspension of PLGA-only or PLGA–vancomycin microparticles (10 mg/mL in ultrapure water) was applied onto each PTFE disc using a fixed pipetted volume and spread manually with a sterile microspatula to cover the surface as uniformly as possible. The discs were allowed to dry under sterile airflow and subsequently dried overnight in a laminar-flow cabinet. Coated discs were stored in sterile containers until implantation (Figure 2). Quantitative assessments of coating thickness, surface coverage, or adhesion strength were not per-formed in this preliminary pilot study. The coating procedure was developed to validate the technical feasibility of applying PLGA-based formulations onto PTFE implant analogs and to enable subsequent short-term in vivo tolerability testing.

### 2.6. In Vivo Surgical Procedure

All animal procedures were approved by the Institutional Animal Ethics Committee (approval no. 366/03.09.2025) and conducted in accordance with Directive 2010/63/EU on the protection of animals used for scientific purposes. Four adult Wistar rats (two males and two females, 400–500 g) were housed under controlled environmental conditions (22 ± 2 °C, 50–60% humidity, 12 h light/dark cycle) with ad libitum access to food and water [22,23]. The number of animals was determined in accordance with ethical guidelines requiring the minimization of animal use in exploratory studies and is consistent with pilot in vivo designs focused on feasibility and early tolerability rather than statistical comparisons.

A bilateral paired implantation design was used to minimize animal use in accordance with 3R principles. Each animal received one PLGA-only-coated disc on the left side (reference implant) and one PLGA–vancomycin-coated disc on the right side (test implant). This design allowed each rat to serve as its own internal control, reducing inter-individual variability and supporting the exploratory nature of this pilot study.

**Figure 3:**
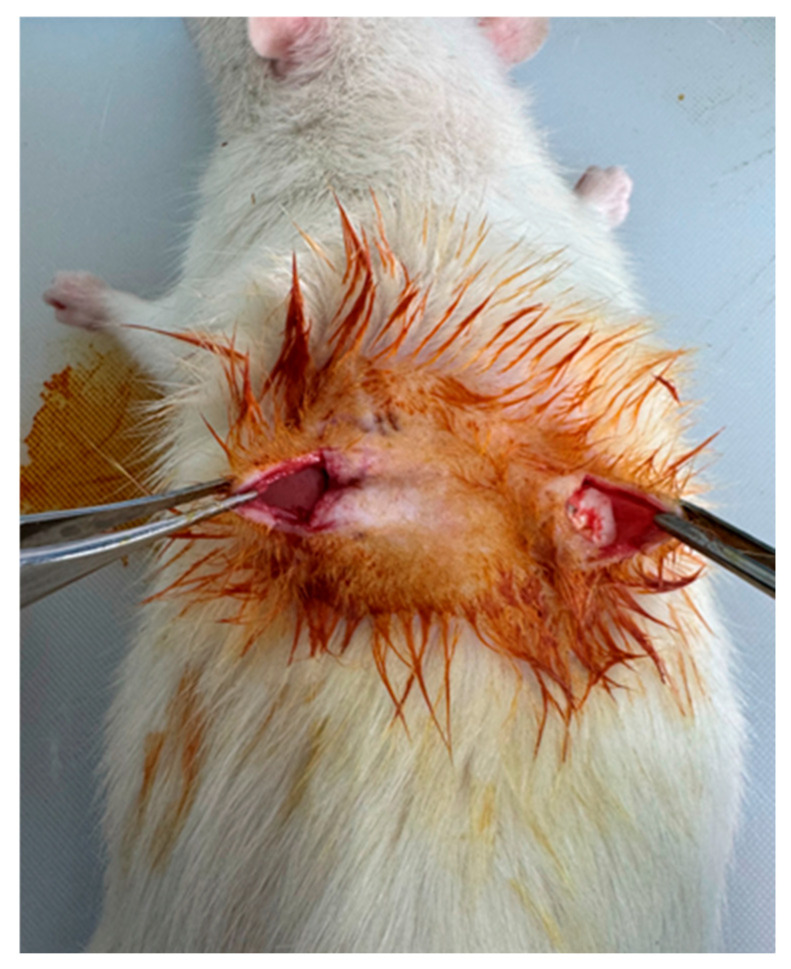
Subcutaneous pockets.

**Figure 4:**
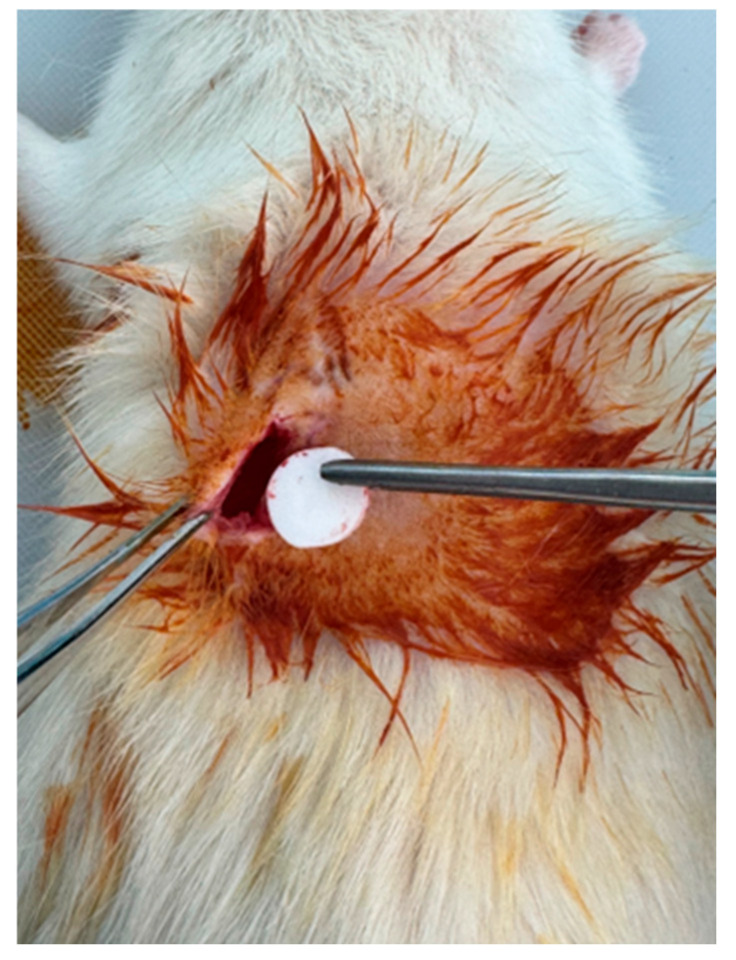
Implant insertion.

Anesthesia was induced via intraperitoneal injection of ketamine (50 mg/kg) and xylazine (5 mg/kg), and anesthetic depth was verified using the toe-pinch test [23]. The dorsal region was shaved and disinfected with 70% ethanol followed by povidone–iodine. Two longitudinal incisions (~1.5 cm) were made approximately 1 cm lateral to the midline, and separate subcutaneous pockets were created via blunt dissection (Figure 3). The reference and test implants were placed into their respective pockets (Figure 4), and the skin was closed with interrupted 4-0 polypropylene sutures. Postoperative analgesia was provided with meloxicam (1 mg/kg, subcutaneously, once daily for two days).

Animals were monitored daily for wound healing, general behavior, grooming, mobility, and food intake. Body weight was recorded every three days. Humane endpoints were predefined according to institutional guidelines and Directive 2010/63/EU, including >15% sustained weight loss, severe lethargy, open or necrotic wounds, purulent discharge, or persistent distress unresponsive to analgesia. No humane endpoints were reached.

A four-week follow-up period was selected based on established rodent subcutaneous implantation models, where early host-tissue responses can be assessed, including the initial stages of capsule formation evaluated primarily through histology. At the endpoint, animals were euthanized via ketamine/xylazine overdose. Implant sites were reopened and examined macroscopically for signs of inflammation, capsule formation, or exudate. Tissue samples corresponding to each implant were harvested for histological evaluation.

The predefined primary endpoint of this pilot study was short-term local tolerability, assessed through macroscopic wound appearance and absence of adverse local reactions. Secondary endpoints included body weight evolution and preliminary histological characteristics at explantation (capsule thickness and inflammatory infiltrate). As this was an exploratory feasibility study, no formal statistical analysis was planned.

### 2.7. Histological Processing and Evaluation

At the end of the 4-week implantation period, tissue samples surrounding each disc were harvested en bloc and immediately fixed in 10% neutral-buffered formalin for 48 h. Specimens were processed routinely through graded alcohols, cleared in xylene, and embedded in paraffin. Sections of 4 µm thickness were obtained using a rotary microtome. Two staining protocols were performed: hematoxylin and eosin (H&E) for general morphology and inflammatory infiltrate and Masson’s trichrome for qualitative visualization of collagen deposition and capsule structure.

Slides were scanned using the Motic EasyScan system, and digital images were viewed and managed using Motic DSAssistant. Quantitative histological analyses were performed using Image-Pro Plus AMS (Media Cybernetics, Rockville, MD, USA). Capsule thickness was measured at three representative points per section using calibrated linear measurement tools. Collagen deposition on trichrome-stained sections was quantified as the percentage of collagen-positive area within the capsule region using standardized threshold-based segmentation. Inflammatory infiltrate was scored semi-quantitatively on H&E-stained sections using a 0–3 scale (0 = absent, 1 = mild, 2 = moderate, 3 = marked).

All histological assessments were conducted under blinded conditions; the evaluator had no information regarding whether samples corresponded to reference (PLGA-only) or test (PLGA–vancomycin) implants.

## 3. Results

### 3.1. Particle Characterization

Dynamic light scattering (DLS) measurements of the vancomycin-loaded PLGA formulation indicated a mean hydrodynamic diameter of approximately 958 nm, based on triplicate technical readings obtained from the same preparation and placing them in the submicron range (nanoparticle–microparticle boundary). The size distribution was unimodal, with a predominant peak near 1 µm. A small proportion of particles below 600 nm was detected, but these did not form a distinct secondary peak and represented a minor fraction of the overall population. The measured polydispersity index (PDI) was 0.301, consistent with the moderate size heterogeneity commonly observed in PLGA particles produced via double-emulsion techniques.

**Figure 5:**
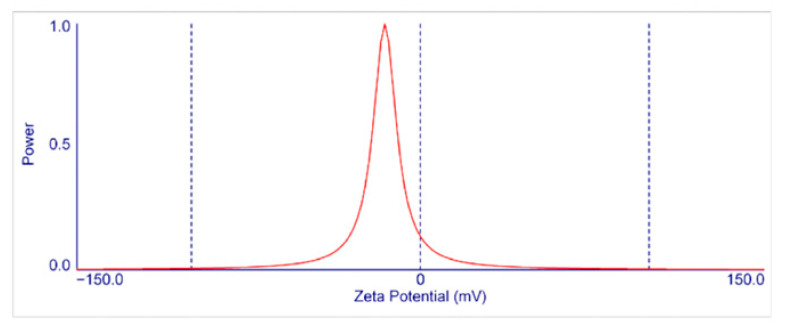
Zeta potential of PLGA–antibiotic particles.

The zeta potential of the PLGA–vancomycin particles was −15.5 ± 2.1 mV (Figure 5), indicating a mildly negative surface charge in aqueous dispersion. No evaluation of long-term colloidal stability or morphology (e.g., SEM) was conducted in this pilot study, as the primary aim was to obtain a workable particle suspension for the coating procedure [24].

Because only a single preparation was analyzed in this preliminary stage, no batch-to-batch variability assessment or additional characterization (e.g., encapsulation efficiency, release profile) was performed. Within the context of this exploratory study, the obtained particle size and charge values were adequate for preparing coated PTFE discs used in the in vivo protocol.

### 3.2. In Vivo Evaluation

All animals recovered uneventfully from anesthesia and displayed normal behavior, feeding, and grooming throughout the 4-week observation period. No wound dehiscence, infection, or purulent discharge was observed. The surgical sites healed primarily within 10–12 days, and sutures were removed after complete epithelialization.

**Figure 6:**
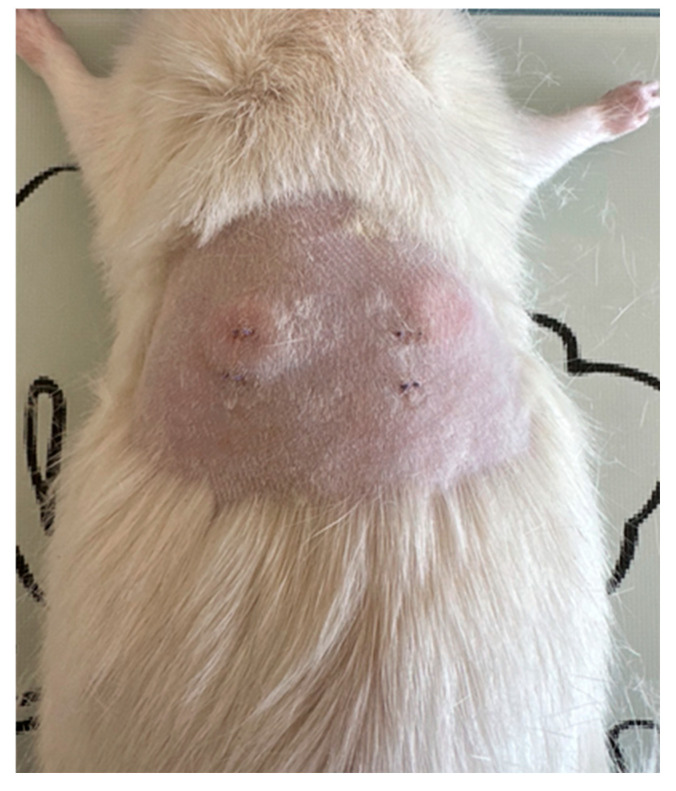
Implantation sites after 4 weeks.

**Figure 7:**
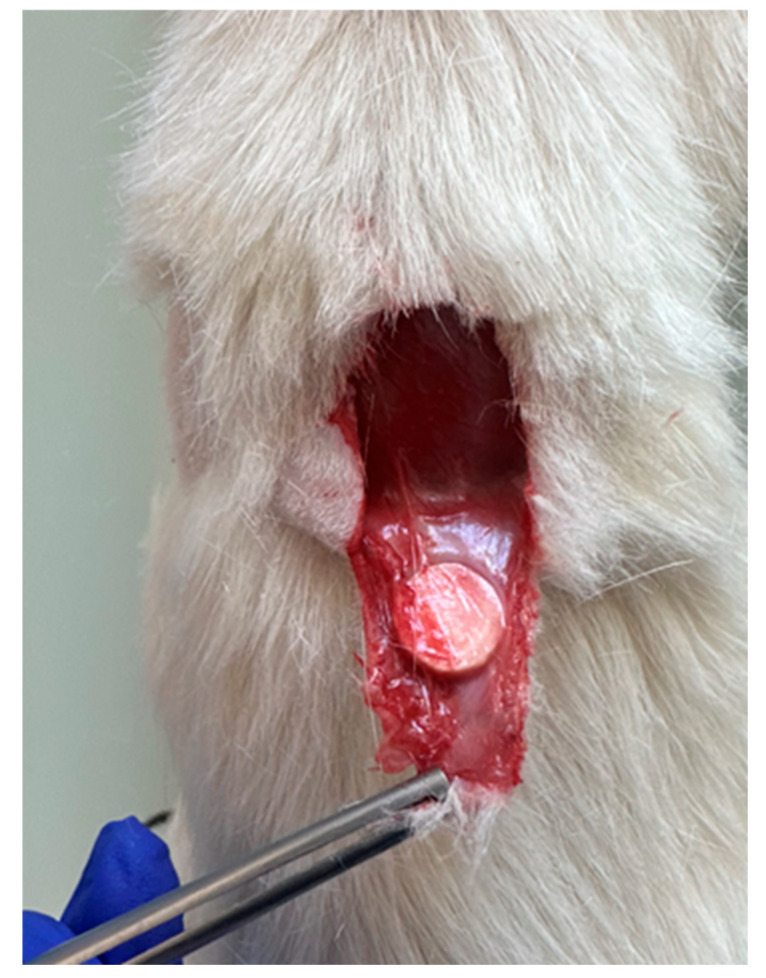
Explantation.

At explantation, both the reference (PLGA-only) and test (PLGA–vancomycin) discs were found in their expected subcutaneous positions. A thin, early fibrous capsule was visible around all implants, consistent with normal foreign-body tissue response at this time point. The surrounding tissues appeared grossly healthy, without macroscopic signs of necrosis, excessive erythema, or purulent exudate. No notable macroscopic differences were observed between the two implantation sites (Figure 6 and Figure 7).

These observations indicate that the surgical procedure and coating application were well tolerated in this pilot model and provide support for the feasibility of the bilateral implantation protocol used in this study [25,26].

### 3.3. Body Weight Evolution

Body weight was monitored every three days as an indicator of general postoperative condition. All animals maintained stable weights throughout the 4-week period, with only minor fluctuations within the normal range for adult Wistar rats. No sustained weight loss (>10–15%) was observed, and no animal met predefined humane endpoint criteria.

**Figure 8:**
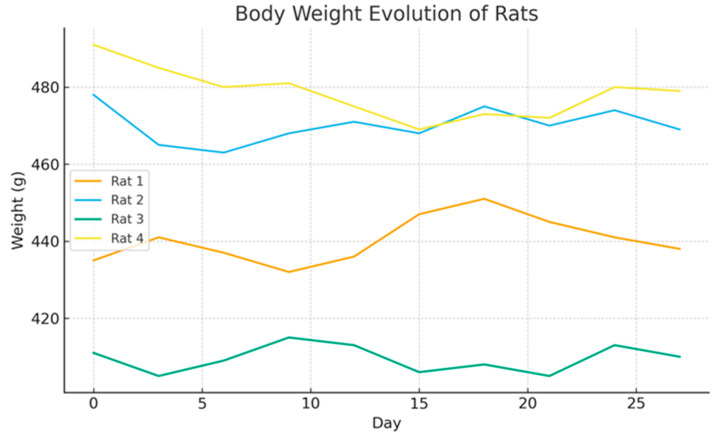
Body weight evolution.

These findings support the absence of systemic distress or adverse postoperative effects in this pilot model (Figure 8).

### 3.4. Histological Findings

**Figure 9:**
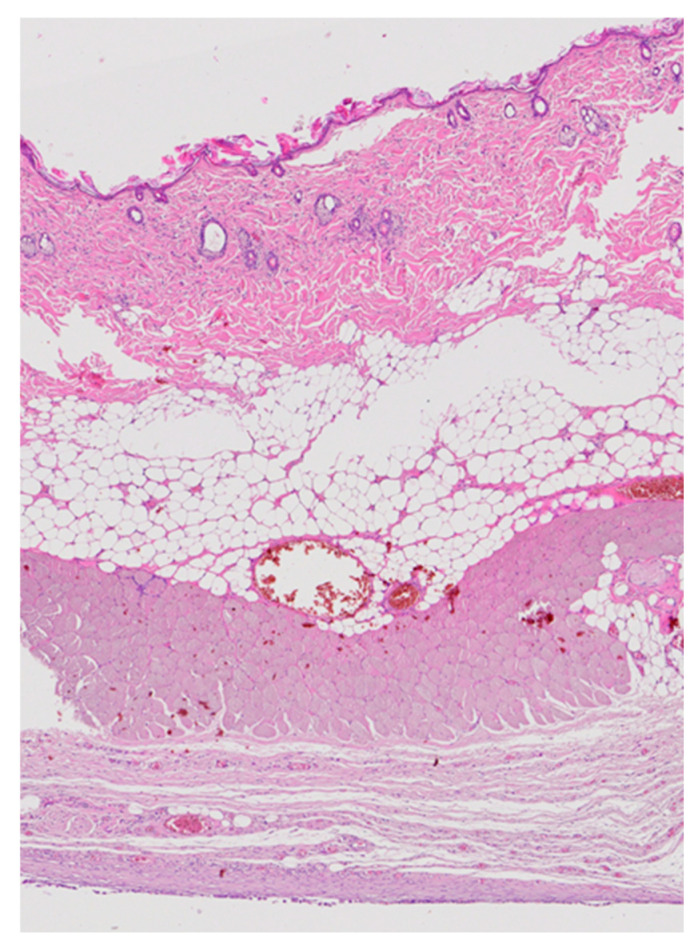
Low-magnification overview of the dorsal implantation site showing skin, subcutaneous tissue, adipose tissue, muscle, and developing fibrous capsule (H&E, 20×).

Low-magnification overview images showed normal architecture of the skin, subcutaneous tissue, and underlying muscle at all implantation sites (Figure 9). Both groups displayed normal epidermal and dermal structures, intact adipose tissue, and well-preserved skeletal muscle fibers. No necrosis, abscess formation, or exuberant inflammatory infiltrate was observed. A thin fibrous capsule surrounded the implant in all specimens, consistent with a minimal foreign-body reaction typical for subcutaneous bio-materials in rodents.

Capsular tissue formed consistently around both PLGA (blank) and PLGA–vancomycin-coated discs.

Based on 20× H&E sections, the mean capsule thickness was:

- PLGA (Blank): 529.61 ± 62.92 µm
- PLGA + vancomycin: 208.89 ± 55.64 µm

**Figure 10:**
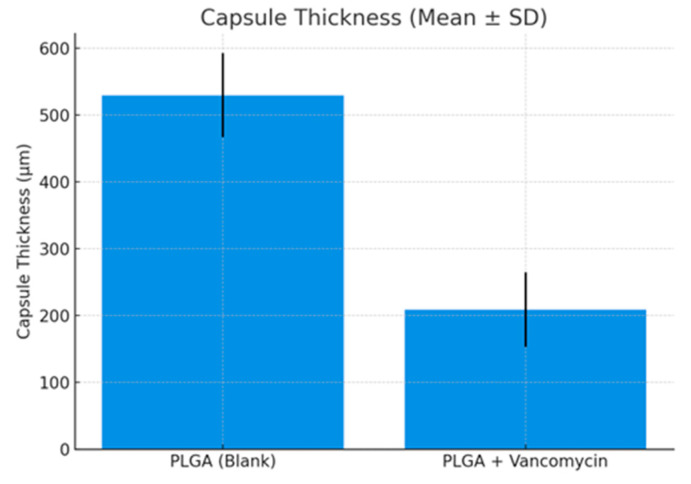
Capsule thickness.

Although numerical differences were observed, this pilot study was not designed or powered for statistical comparison; therefore, these values are presented descriptively (Figure 10). The capsules were thin, well-organized, and composed of parallel collagen bundles with scattered fibroblasts, without evidence of excessive fibrosis.

Quantitative analysis of trichrome-stained sections (collagen IOD per standardized area) showed:

- PLGA (blank): 8.68 × 10^7^ ± 2.76 × 10^7^ pixels
- PLGA + vancomycin: 4.36 × 10^7^ ± 3.08 × 10^6^ pixels

**Figure 11:**
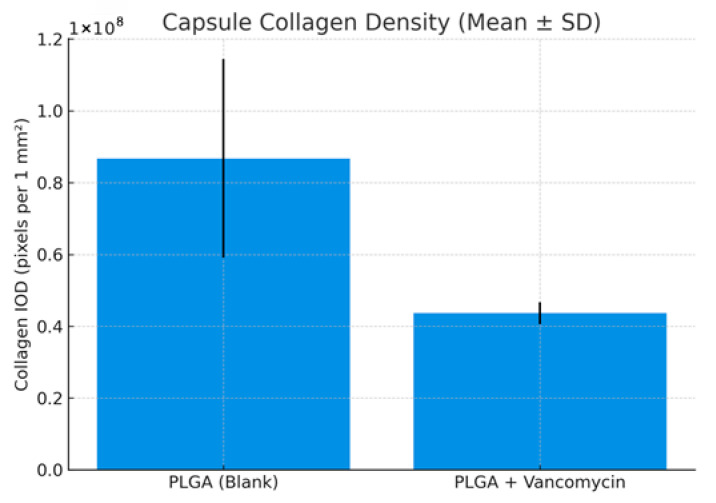
Capsule collagen density.

Both groups demonstrated moderate collagen deposition consistent with early capsule formation at four weeks. No areas of dense, hypertrophic fibrosis or disorganized collagen bundles were identified. The bar chart representation is provided in Figure 11.

Inflammatory infiltrate was assessed semi-quantitatively on H&E-stained sections (0–3 scale), evaluating lymphocytes, macrophages, neutrophils, and multinucleated giant cells.

The findings were as follows:

- PLGA (blank): mild inflammatory infiltrate (score 2) with scattered lymphocytes and macrophages, typical of early foreign-body reaction.
- PLGA + vancomycin: very mild infiltrate (score 1), with sparse inflammatory cells and no giant-cell reaction.

**Figure 12:**
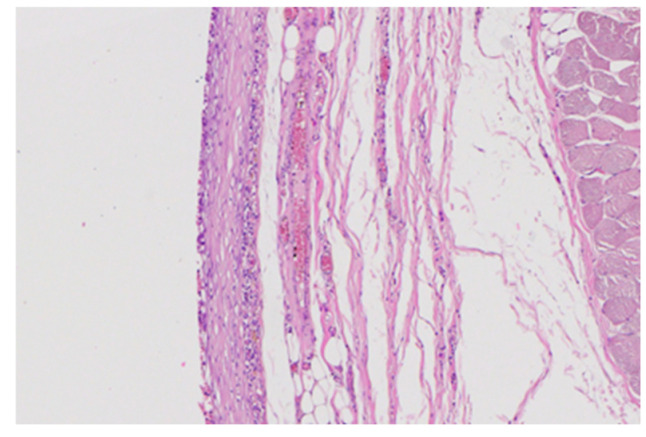
Representative H&E image of the fibrous capsule around PLGA (blank) implant showing moderate inflammatory infiltrate (score 2). Magnification: 20×.

**Figure 13:**
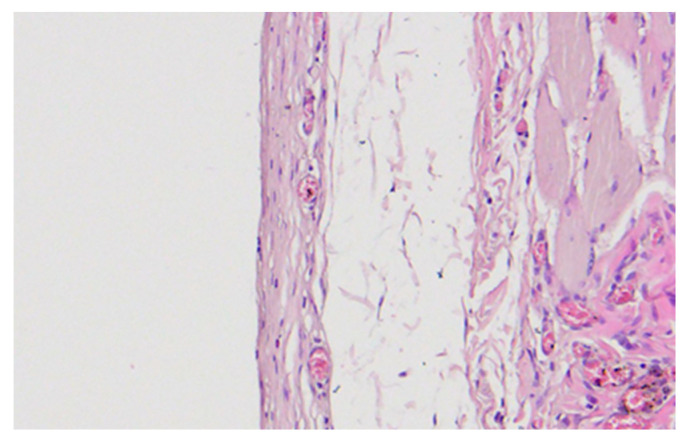
Representative H&E image of the fibrous capsule around PLGA + vancomycin implant showing minimal inflammatory infiltrate (score 1). Magnification: 20×.

Representative images are shown in Figure 12 and Figure 13.

No signs of granulomatous inflammation, foreign-body giant cell clusters, abscesses, or tissue destruction were noted in any sample.

These findings support short-term local tolerability of both PLGA and PLGA–vancomycin coatings in this rat model.

## 4. Discussion

Implant-associated infections remain a major challenge in reconstructive and aesthetic breast surgery. Local antibiotic delivery systems are increasingly explored to overcome the limited penetration of systemic agents at the implant–tissue interface [7,8] and may help reduce postoperative complications and implant failure [27,28]. PLGA-based drug delivery platforms offer tunable degradation profiles and controlled release properties, and they have been successfully applied across multiple biomedical fields [29,30].

In this pilot study, we developed a PLGA–vancomycin sub-micron particle formulation and demonstrated its feasibility for coating inert implant-like substrates. Comparable coating strategies have been described in other implant models, including dental applications, highlighting their translational potential [31]. The sub-micron particles obtained through the double-emulsion solvent-evaporation method showed size and surface-charge characteristics consistent with published PLGA formulations used for controlled antibiotic delivery. However, only basic characterization was performed in this first phase; advanced physicochemical analyses such as release kinetics, encapsulation efficiency, and morphology will be included in subsequent studies.

Although PTFE was used as a practical surrogate for smooth silicone implants, the two materials are not identical. They share advantageous features for rodent models—chemical inertness, smooth surfaces, and ease of handling—but they differ in surface chemistry, hydrophobicity, stiffness, and long-term behavior. These factors may influence biological responses and biofilm formation in clinical scenarios. Therefore, while PTFE was suitable for this preliminary feasibility model, future studies will apply the coating directly onto medical-grade silicone and include detailed surface characterization.

The in vivo component of this study focused strictly on short-term local tolerability. All animals recovered normally, and no signs of infection or adverse local reactions were observed during the four-week follow-up. The bilateral implantation model allowed each animal to serve as its own internal control, minimizing interindividual variability and reducing the total number of animals in accordance with the 3R principles. The absence of macroscopic inflammation aligns with previous reports describing low tissue reactivity of PLGA-based systems [2,8].

Preliminary histological observations showed typical early foreign-body capsule formation with modest collagen deposition and a mild inflammatory infiltrate—findings consistent with expected early responses in subcutaneous rodent models. These results should not be interpreted as a full biocompatibility profile but rather as early evidence of short-term tolerability. Quantitative histology, immunohistochemistry, cytokine analysis, and long-term evaluation will be required to define the biological response in detail.

Photographs included in the manuscript serve only as visual documentation of surgical procedures and were not intended as analytical endpoints. Likewise, this pilot stage did not include drug loading quantification, release profiles, or antimicrobial assays; these limitations are acknowledged and will be addressed in planned follow-up studies.

Overall, this study establishes a practical and ethically refined small-animal model for early testing of PLGA-based coatings on implant-like substrates. Building upon these preliminary findings, future work will focus on detailed material characterization, drug-release analysis, antibacterial efficacy, and the development of multifunctional coatings capable of modulating the immune and fibrotic responses at the implant–tissue interface [32,33].

## 5. Conclusions

This pilot study demonstrates the feasibility of applying vancomycin-loaded submicron PLGA particles as a surface coating on PTFE implant analogs and confirms that the coated implants were well tolerated in the short term. The bilateral rat model proved practical, reproducible, and ethically refined, offering a straightforward platform for early assessment of local tissue tolerability. Preliminary histological findings showed typical early foreign-body responses without evidence of excessive inflammation or adverse reactions.

These conclusions are intentionally limited to feasibility and short-term tolerability, as the present work did not evaluate vancomycin loading, drug-release kinetics, antibacterial performance, or long-term tissue responses. However, establishing a functional coating method and an in vivo pilot model represents a necessary first step toward more comprehensive analyses.

Building upon this foundation, future studies will expand the coating approach to medical-grade silicone, incorporate detailed physicochemical and release characterization, and include antibacterial and infection-challenge models. Larger cohorts, additional bioactive compounds, and extended histological and molecular analyses will support the development of next-generation multifunctional implant coatings with improved therapeutic potential.

## Data Availability

The data presented in this study are available upon request from the corresponding author.

## Abbreviations

The following abbreviations are used in this manuscript:

DCM	Dichloromethane
DLS	Dynamic Light Scattering
FDA	Food and Drug Administration
MRSA	Methicillin-Resistant *Staphylococcus aureus*
PDI	Polydispersity Index
PLGA	Poly(lactic-co-glycolic acid)
PTFE	Polytetrafluoroethylene
PVA	Poly(vinyl alcohol)
TGF-β1	Transforming Growth Factor Beta 1
TNF-α	Tumor Necrosis Factor Alpha
IL-6	Interleukin 6
W1/O/W2	Water-in-Oil-in-Water (double emulsion)
